# Whole blood gene expression analysis of spontaneous hypertriglyceridemia in dogs suggests an underlying pro-thrombotic process

**DOI:** 10.1371/journal.pone.0313343

**Published:** 2024-11-12

**Authors:** Lauren A. Baker, Katie M. Minor, Nicole Tate, Eva Furrow

**Affiliations:** 1 Department of Animal and Dairy Sciences, University of Wisconsin-Madison, Madison, Wisconsin, United States of America; 2 Department of Veterinary Clinical Sciences, College of Veterinary Medicine, University of Minnesota, St. Paul, Minnesota, United States of America; University of Minnesota, UNITED STATES OF AMERICA

## Abstract

Hypertriglyceridemia (HTG) is influenced by multiple genetic and environmental factors. Spontaneous, idiopathic HTG is common in the Miniature Schnauzer dog and presumed to have a strong genetic influence in this breed. To define genes that are differentially expressed in dogs with HTG, we performed RNA sequencing on peripheral blood of 13 Miniature Schnauzers with HTG and 18 controls. We identified 110 differentially expressed genes (DEGs). Pathway analysis suggests an ongoing pro-thrombotic, endothelial activation process in dogs with HTG. The gene with the largest fold change (5.4 ± 1.4, P_adj_ = 4.4E-04), *SERPINE1*, encodes plasminogen activator inhibitor 1 (PAI-1), a known risk factor for atherosclerosis and thrombosis. Other top DEGs, including *SHANK3*, *MMRN1*, and *FZD7*, are involved in endothelial activation. Two of the top DEGs, *ARHGAP29* and *ARHGAP21*, inhibit pro-thrombotic pathways and are potentially protective of disease sequelae. Top DEGs, including *SERPINE1* and *ARHGAP21*, have also been linked to metabolic syndrome or its features (e.g. insulin resistance) in humans and animal models. Our findings indicate that HTG in the Miniature Schnauzer dog has similar features to HTG and metabolic syndrome in humans, highlighting the potential use of the dog as a spontaneous model for further research into the etiology and effects of HTG.

## Introduction

Hypertriglyceridemia (HTG) is a common abnormality encountered in clinical practice with an estimated population prevalence of 26% of adults in the United States [1]. Hypertriglyceridemia is associated with several ancillary health conditions including atherosclerotic cardiovascular disease, type 2 diabetes, fatty liver disease, and pancreatitis, among others [2, 3]. Traditionally, the HTG diagnosis has been divided into primary and secondary types, with primary HTG understood to have a genetic cause and secondary HTG considered a consequence of another disease process or medication [2]. However, there is increasing recognition that HTG is a complex and heterogeneous disorder that is influenced by multiple overlapping genetic, environmental, and lifestyle factors [4]. This includes patients with familial HTG, previously referred to as type IV familial dyslipidemia, which is characterized by increased secretion of very low-density lipoprotein (VLDL) particles from the liver [5]. While severe HTG (>10 mmol/L [885 mg/dL]) is more likely to be monogenic than mild-to-moderate HTG, most HTG cases, regardless of severity, are estimated to be polygenic [6–8].

Hypertriglyceridemia also spontaneously presents in dogs. There is a particularly high prevalence in Miniature Schnauzer dogs, with greater than 75% of the breed affected by 10 years of age [9]. A strong genetic predisposition to HTG is presumed to exist in this breed [9–11]. In Miniature Schnauzers, HTG is typically characterized by increases in triglyceride-rich lipoproteins (VLDL with or without increased chylomicrons) [12], making it similar to familial HTG in humans. However, some Miniature Schnauzers also have elevations in low density lipoprotein (LDL) fractions [13]. The development of HTG occurs with increased triglyceride production, decreased clearance, or both [4]. While lipoprotein lipase (LPL) activity is decreased in Miniature Schnauzers with HTG, supporting decreased triglyceride clearance, the decrease is relatively modest and not thought to be the sole cause of HTG [14]. Additional pathways impacting triglyceride production or clearance are likely involved. Further, genetic investigations of the top candidate genes for HTG have not identified a monogenic cause in Miniature Schnauzers [10, 11]. This suggests that the mechanisms contributing to HTG in Miniature Schnauzers are multifactorial, similar to in humans.

Like humans, HTG in dogs is associated with pancreatitis, hepatobiliary disease, and insulin resistance [15–17]. Unlike humans, atherosclerotic cardiovascular disease is uncommon in dogs because dogs have relatively low LDL levels due to absence of active cholesterol ester transfer protein (CETP), an enzyme responsible for moving cholesterol and triglycerides between various lipoproteins [18]. Even with this difference, we propose the Miniature Schnauzer dog as a useful natural model to study contributions to and consequences of HTG.

A growing body of evidence suggests that the underlying genetic basis of polygenic conditions like HTG lies not in gene mutations, but in transcriptional regulation of the genes that drive disease processes [19]. The purpose of the present investigation was to define genes that are differentially expressed in the dog model of HTG. We performed RNA sequencing (RNA-seq) of peripheral blood from Miniature Schnauzer dogs with and without HTG. We identified differentially expressed genes that clearly stratify cases from controls. The top genes suggest an ongoing pro-thrombotic, endothelial activation process in dogs with HTG. Several of these genes are also associated with insulin resistance. We believe these findings position the Miniature Schnauzer dog as a spontaneous model for the study of HTG and add to data on how the transcriptome changes with HTG, which may ultimately aid development of treatments that benefit both animal and human health.

## Results

We performed RNA-seq on 31 purebred Miniature Schnauzer dogs, including 13 HTG cases and 18 controls (Table 1). This sample size comprised all dogs that met the recruitment criteria and had an available biobanked RNA sample. Eight of 13 cases and 10 of 18 controls were male (P = 1.00). All males had been castrated, and all but one female had undergone ovariohysterectomy surgery. The average age of cases and controls was 10.2 ± 1.4 and 8.9 ± 1.6 years, respectively (P = 0.21). Median body condition score (BCS) was 6 (range 4–7) in the cases and 5 (range 3–7) in the controls (P = 0.33). Because hyperlipidemia can occur secondary to endocrinopathies, dogs were screened for endocrinopathies during the recruitment process. All HTG cases tested negative for diabetes mellitus, hypothyroidism, and hypercortisolism (screened with a urine cortisol-to-creatinine ratio in 12 cases and a low dose dexamethasone suppression test in 1 case). The 31 study dogs were consuming 30 different commercial diets; 2 controls resided in the same household and were fed the same diet. Median dietary fat content was 4.2 ± 1.2 g/100 kcal in cases and 3.9 ± 0.8 g/100 kcal in the controls (P = 0.64); dietary fat could not be determined for 5 dogs due to inability to obtain manufacturer nutrient information (n = 4) or highly varied daily diet (n = 1). Blood lipid values are presented in Table 1. The median triglyceride concentrations were 578 (IQR 494) mg/dL for cases and 65 (IQR 30) mg/dL for controls. The median cholesterol concentrations were 301 (IQR 109) mg/dL for cases and 172 (IQR 70) mg/dL for controls. Only three dogs with HTG had elevated cholesterol levels, consistent with the expected presentation of HTG in Miniature Schnauzer dogs [9]. All 18 controls screened negative for diabetes mellitus, and 15 controls screened negative for both hypothyroidism and hypercortisolism.

**Table 1:** Blood lipid values of hypertriglyceridemic case and normotriglyceridemic control Miniature Schnauzer dogs at the time of RNA sample collection.

Dog ID	Age (years)	Sex^a^	BCS^b^	Dietary Fat (g/100 kcal)	Triglycerides (mg/dL)^c^	Cholesterol (mg/dL)^d^
Case_01	11	FS	6	3.9	2392	NA
Case_02	10	MN	7	6.2	1164	457
Case_03	9	MN	6	NA	973	428
Case_04	12	MN	7	4.8	854	322
Case_05	9	MN	6	5.3	720	185
Case_06	11	MN	4	5.5	618	403
Case_07	9	FS	6	4.3	578	302
Case_08	8	FS	6	NA	431	299
Case_09	10	MN	6	4.0	393	320
Case_10	9	MN	5	NA	360	257
Case_11	14	MN	5	2.9	334	243
Case_12	10	FI	5	2.2	271	148
Case_13	10	FS	5	3.3	263	206
Control_01	8	FS	4	5.0	93	277
Control_02	8	FS	5	3.7	88	369
Control_03	10	MN	6	3.9	88	NA
Control_04	9	MN	7	3.6	85	136
Control_05	10	MN	5	NA	76	155
Control_06	7	FS	6	3.3	75	203
Control_07	10	MN	6	4.0	74	172
Control_08	10	FS	7	6.5	66	138
Control_09	9	MN	7	3.7	66	145
Control_10	8	FS	5	NA	63	226
Control_11	9	FS	6	3.6	61	190
Control_12	11	FS	5	4.0	52	NA
Control_13	9	MN	5	3.4	50	227
Control_14	9	MN	4	4.2	44	162
Control_15	9	MN	3	3.9	43	184
Control_16	10	FS	4	3.9	41	NA
Control_17	13	MN	6	3.6	37	132
Control_18	11	MN	4	4.2	35	145

^*a*^MN = male neutered; FS = female spayed; FI = female intact.

^*b*^BCS = body condition score, grading on a 1–9 scale (1 = emaciated, 9 = obese)

^*c*^The laboratory reference interval for triglycerides in dogs is 26–108 mg/dL.

^*d*^The laboratory reference interval for cholesterol in dogs is 143–373 mg/dL.

**Fig 1:**
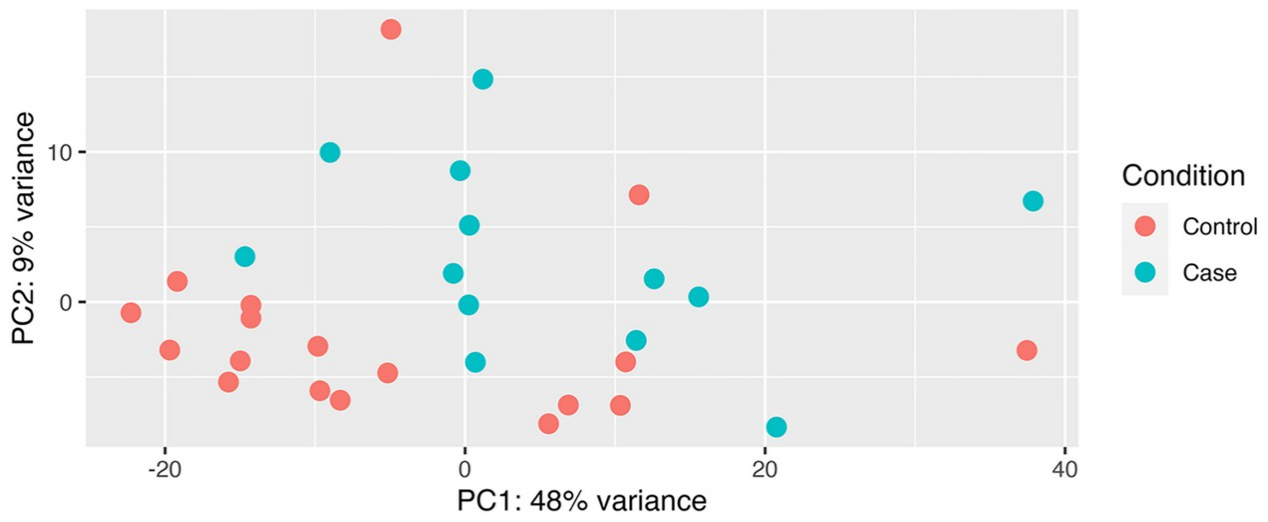
PCA plot of RNA-seq data from hypertriglyceridemic case and normotriglyceridemic control Miniature Schnauzer dogs.

**Fig 2:**
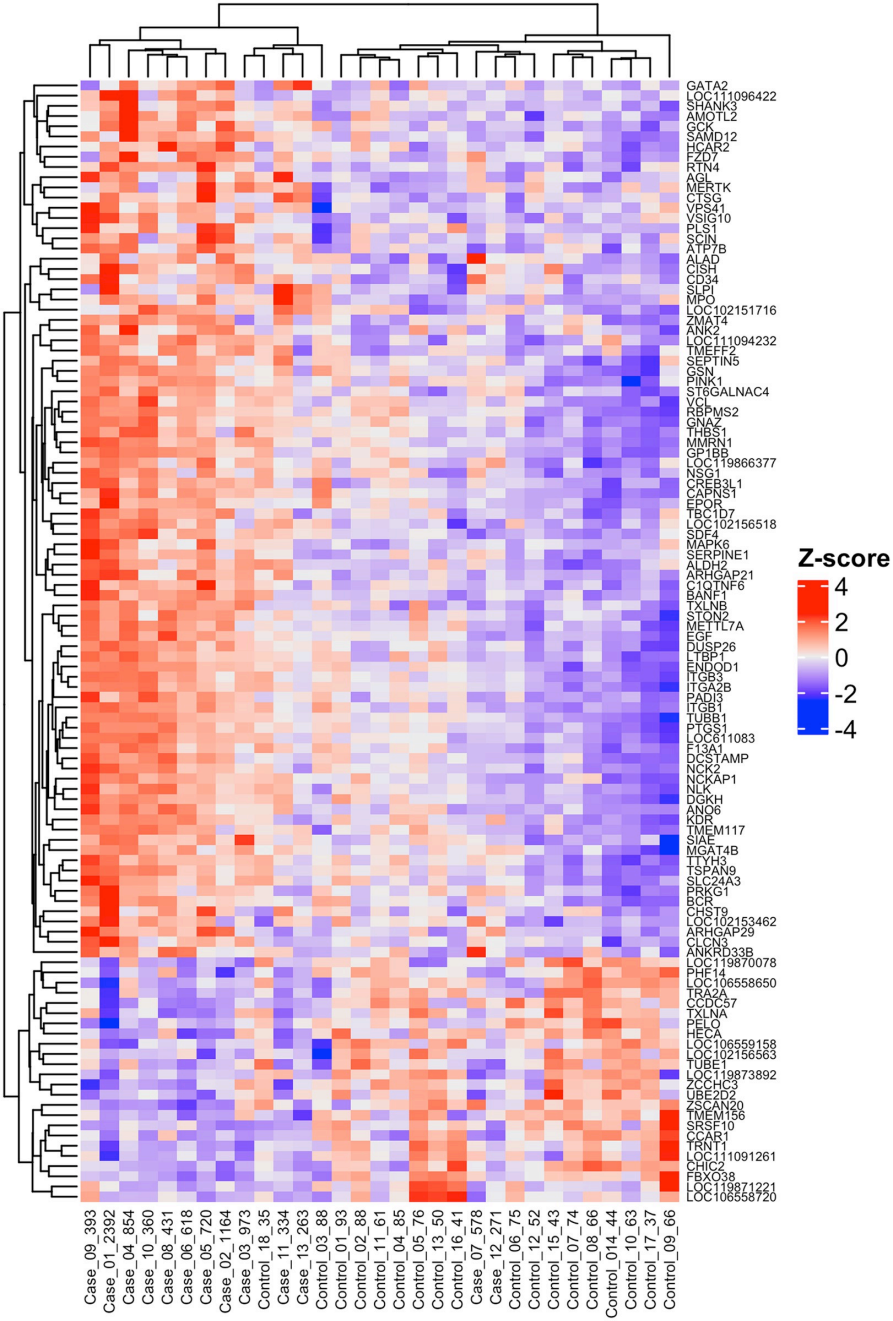
Hierarchical clustering of 110 differentially expressed genes in Miniature Schnauzers with and without hypertriglyceridemia. Individual dogs are labeled DogID_TriglycerideConcentration (mg/dL).

All RNA samples were determined to be good quality after FastQC analysis. On average, there were 125,789,945 reads per sample (62,894,972 pairs), with 100% of reads properly paired. The average primary and secondary alignment percentages were 55.7 and 0.2%, respectively (S1 Table in S1 File). Results of principal components analysis using the top 500 most variable genes are shown in Fig 1. After adjustment for multiple testing, there were 110 genes that were differentially expressed between case and control groups (S2 Table in S1 File). Hierarchical clustering of differentially expressed gene (DEG) counts revealed a clear pattern of expression in case vs. control groups (Fig 2). Generally, cases and controls clustered in their groups with three exceptions: case_07 and case_12 clustered with controls, and control_18 clustered with cases. The top ten DEGs are listed in Table 2 and plotted in S1 Fig in S1 File. All ten genes were expressed more highly in cases than in controls. Confirmatory RT-qPCR for the top two genes validated differential expression for *ARHGAP29* (P = 2.6E-04, relative fold expression = 4.5) and *SERPINE1* (P = 4.5E-04, relative fold expression = 4.34), S2 Fig in S1 File.

**Table 2:** Top 10 differentially expressed genes from DESeq2 analysis of hypertriglyceridemic case and normotriglyceridemic control Miniature Schnauzer dogs.

Gene	baseMean	log2FoldChange	lfcSE	Statistic	P-value	P_adj_
*ARHGAP29*	73.3	2.02	0.34	5.90	3.69E-09	5.20E-05
*SERPINE1*	31.9	2.42	0.45	5.34	9.19E-08	4.40E-04
*ARHGAP21*	569.9	0.51	0.10	5.29	1.24E-07	4.40E-04
*SHANK3*	126.3	1.60	0.30	5.29	1.25E-07	4.40E-04
*MMRN1*	606.5	1.14	0.22	5.14	2.75E-07	7.77E-04
*ST6GALNAC4*	1524.7	0.65	0.13	5.06	4.13E-07	9.71E-04
*FZD7*	40.5	1.21	0.24	4.99	5.94E-07	1.20E-03
*STON2*	3905.2	0.66	0.14	4.78	1.73E-06	2.75E-03
*RTN4*	1690.7	0.21	0.04	4.78	1.76E-06	2.75E-03
*SLPI*	17.5	1.91	0.41	4.64	3.46E-06	4.88E-03

^a^baseMean = mean of normalized counts for all samples; lfcSE = standard error of log2 fold change; Statistic = Wald test statistic; P-value = Wald test P-value; P_adj_ = Benjamini-Hochberg adjusted P-values.

Enrichr analysis of Reactome pathways revealed a significant enrichment of 9 biological pathways with the top 4 related to platelet function and activation (S3 Table in S1 File). Analysis with BioPlanet identified 3 top pathways related to platelets and highlighted 10 additional pathways (S4 Table in S1 File).

## Discussion

Through RNA sequencing of whole blood, this study identified genes that are up- or down-regulated in Miniature Schnauzer dogs with HTG relative to those with normal serum triglyceride concentrations. These DEGs and the biological pathways they participate in are consistent with ongoing platelet and endothelial activation and might provide direction on the underlying pathogenesis and consequences of HTG in the Miniature Schnauzer dog. The top DEGs are also implicated in human HTG and metabolic syndrome patients, demonstrating potential of the dog as a spontaneous model to inform the understanding of these diseases in humans.

The top DEGs in this study are consistent with an ongoing pro-thrombotic endothelial activation process and biological attempts to regulate that process in dogs with HTG. Top Reactome and BioPlanet pathways involved platelet function and activation. The DEG with the largest fold change, *SERPINE1*, encodes plasminogen activator inhibitor 1 (PAI-1). This DEG showed a >5-fold increased expression in HTG cases compared to controls in the RNA-seq data, and >4-fold increased expression with confirmatory RT-qPCR. PAI-1 is the major physiological inhibitor of fibrinolysis and plays a role in multiple other biological processes including degradation of extracellular matrix, wound-healing, angiogenesis, and inflammation [20]. Elevated levels of PAI-1 create a pro-thrombotic state that has been linked to atherosclerosis and cardiovascular disease more generally [21]. The increased expression detected in the present study is likely a consequence of HTG. VLDL is capable of directly increasing levels of PAI-1 through a VLDL response element located in the promotor region of the *SERPINE1* gene [22, 23], supporting a pro-thrombotic state. Dogs that are hyperlipidemic or dyslipidemic secondary to diabetes mellitus or hypercortisolism also have increased plasma PAI-1 activity [24]. PAI-1 additionally maintains activation of the RhoA/Rho kinase (ROCK) signaling pathway [25]. RhoA/ROCK activation contributes to cytoskeletal rearrangements of endothelial cells and platelets and can thereby result in dysregulation of vascular tone and platelet aggregation [26, 27].

Whether or not increased *SERPINE1* expression effectively increases risk for thrombosis in dogs with HTG is difficult to predict, as these dogs also had increased expression of genes that might protect against thrombosis. The top DEG in this study, *ARHGAP29*, and the third DEG, *ARHGAP21*, encode Rho GTP-ase activating proteins 29 and 21, respectively. These proteins are both inhibitors of the RhoA/ROCK pathway. *ARHGAP29* interacts with Rasip-1 protein to control endothelial barrier function, inhibiting Rho-mediated stress fiber formation and tightening cell-cell junctions [28]. In addition to impacts on endothelial barrier function, *ARHGAP21* shows anti-thrombin activity. *ARHGAP21* is upregulated during megakaryocytic differentiation, and *ARHGAP21-*deficient mice show enhanced hemostasis due to increased platelet size and aggregation [29]. RhoA was detected in our data, but its expression was not significantly different between groups. However, the present study design cannot determine whether the RhoA/ROCK pathway has overall increased, decreased, or unchanged activation in dogs with HTG, as this is affected by post-transcriptional regulation (e.g., phosphorylation) [30].

Other top HTG-associated DEGs in this study have a role in endothelial activation. *SHANK3* (SH3 and multiple ankyrin repeat domains 3) is a scaffolding protein most known for its role in the proper development and function of synapses in the central nervous system. However, it is also expressed in endothelial cells [31] and may play a role in the endothelial activation process indirectly through its interaction with the β-catenin signaling pathway [32]. *MMRN1* (multimerin-1) is found in both platelets and endothelial cells and supports platelet adhesion through fibrillar collagen binding [33]. *FZD7* (frizzled-7) is expressed on the surface of endothelial cells and mediates Wnt/β-catenin signaling, controlling vascular permeability and activating endothelial cell signaling through the disheveled (Dvl) pathway [34, 35]. *RTN4* (Reticulon 4) encodes Nogo-B protein, which may be protective from the formation and progression of atherosclerotic plaques [36]. *SLP1* (secretory leukocyte peptidase inhibitor) is a serine protease inhibitor with anti-inflammatory, tissue-protective effects that has been investigated as a potential gene therapy target for atheroma treatment [37].

Several of the top DEGs are also implicated in metabolic syndrome or its features. The three top HTG-associated Reactome pathways identified here are enriched in metabolic syndrome and non-alcoholic fatty liver disease (NAFLD) in humans [38], and PAI-1 is a biomarker for metabolic syndrome [39]. It has long been recognized that PAI-1 levels are highly correlated with obesity, triglyceride concentrations, insulin resistance, and obesity-related lipid abnormalities [40, 41], though it had been unclear whether this is a cause or a consequence of metabolic syndrome [39]. A recent investigation suggests PAI-1 is directly involved in the development of diet-induced hyperlipidemia and hepatic steatosis through regulation of *PCSK9* (proprotein convertase subtilisin/kexin type 9), an important regulator of lipid metabolism in the liver [42]. *PCSK9* expression was not detected in our samples, likely due to the sample type (peripheral blood rather than liver). Patients with metabolic syndrome show higher RhoA/ROCK activity [43], which again might be a consequence of increased PAI-1. As discussed above, the overexpression of *ARHGAP29* and *ARHGAP21* may be compensatory to prevent excessive RhoA/ROCK activity, but there are potentially deleterious effects that their enhanced expression could have in dogs with HTG. Specifically, *ARHGAP21* expression is permissive to the development of diet-induced decreases in insulin sensitivity [44]. Increased serum insulin and homeostasis model assessment scores occur in Miniature Schnauzers with HTG [45], though they were not measured in the dogs in the present study. This data suggests that the Miniature Schnauzer might serve as a model for investigating the interplay between HTG, PAI-1, the RhoA/ROCK pathway, and insulin resistance.

The sample used for this study has several limitations including lack of age- and sex-matching between case and control groups, restriction to a single breed, and a small sample size. Though statistically significant differences were not detected in age or sex between groups, there were slight imbalances present. Both age and sex can affect gene expression in dogs [46, 47]. All dogs in this study were Miniature Schnauzers, which is advantageous for discovery research but may limit the generalizability of these findings to other dog breeds or species. There is a range of lipoprotein profiles observed in Miniature Schnauzers with HTG [13, 48]. While reduced VLDL clearance due to decreased LPL activity is likely a contributing mechanism [14], additional mechanisms likely exist that further decrease triglyceride clearance or increase their production. Therefore, the present study might not have captured the full spectrum of HTG mechanisms and phenotypes within Miniature Schnauzers.

When interpreting results, it is important to consider the use of blood for RNA-seq. Many of the major genes implicated in HTG pathogenesis, such as LPL, hepatic lipase, and multiple lipoproteins, are primarily expressed in other tissues such as adipose, liver, or intestines [49]. Performing RNA-seq of these other relevant tissues could provide valuable additional insight into underlying pathways involved in HTG in Miniature Schnauzers. A limitation of RNA-seq in general is that it cannot discern underlying mechanism from physiological response. Further, cell concentrations can change in response to environmental stimuli, such as exercise or diet [50, 51]. Though all animals were fasted before blood collection and were recruited through a single study site, the dogs were client owned, and the study did not standardize diet, exercise, or living conditions. Median dietary fat and body condition score were similar between groups, but there might have been other dietary or environmental differences between dogs that confounded results. In humans, consumption of a low-glycemic-index diet and an athletic lifestyle are associated with lower PAI-1 concentrations [52, 53]. Validation in a larger, independent sample is needed.

Existing animal models for studying hyperlipidemic disorders are either induced through single gene manipulations or substantial alterations to a standard diet [54]. These experimental conditions are unlikely to capture all biological processes that contribute to pathogenesis of HTG. Hypertriglyceridemia is exceedingly common in Miniature Schnauzers. Because these animals are pets, there is interest from owners and veterinarians to identify the cause of the disorder, recognize its complications, and develop treatments. Recognition of pet dogs as valuable models for human complex genetic disorders has increased dramatically over the past decade, with multiple consortium efforts to gather environmental and multi-omic data for research currently underway [55–57]. The results of this investigation of the blood transcriptome support the Miniature Schnauzer with HTG as a spontaneous animal model with features similar to humans with HTG and metabolic syndrome. Opportunities for future work in this model include detailed epidemiological investigations, genomic studies, lipidomic analysis, and clinical trials of dietary or pharmacological interventions. Discoveries made in the spontaneous canine model of HTG could then be translated to advance human health.

## Materials and methods

All procedures were performed with approval from the University of Minnesota Institutional Animal Care and Use Committee (protocols #1207A17243 and #1509-33019A). This study is reported in accordance with ARRIVE guidelines. Purebred Miniature Schnauzer dogs were recruited through the University of Minnesota Veterinary Medical Center between March of 2015 and March of 2019. Owner written informed consent was obtained for each dog prior to participation in the study. Owners were requested to withhold food from dogs for 12–18 hours before the scheduled study appointment time. Blood was collected from the jugular vein on awake animals under minimal physical restraint, in accordance with IACUC guidelines. Blood samples were collected for serum biochemistry, endocrine testing, and RNA-seq. Samples for RNA-seq were collected using Tempus blood RNA tubes (ThermoFisher Scientific, Wilmington, DE). Serum biochemistry, including serum triglyceride and cholesterol concentrations, was measured using an AU480 chemistry analyzer (Beckman Coulter Diagnostics, Brea, CA).

Recruitment criteria for the dogs included in this study has been reported previously [58]. All recruited dogs were greater than 7 years of age. Dogs with fasting serum triglyceride concentrations >250 mg/dL were classified as HTG cases [11]. Dogs with fasting serum triglyceride concentration ≤108 mg/dL were classified as controls [9]. Dogs with triglyceride concentrations of 109–250 mg/dL were not included in this study. Dogs were also not included if they had a history or clinical suspicion of an endocrinopathy or were taking medications known to alter lipid levels (e.g., glucocorticoids, phenobarbital, potassium bromide, fibrates, or statins). All HTG cases were screened for disorders that are potential secondary causes of hyperlipidemia, including diabetes mellitus (fasting serum blood glucose concentration), hypothyroidism (serum total thyroxine concentration), hypercortisolism (low dose dexamethasone suppression test or urine cortisol-to-creatinine ratio), and kidney disease (serum creatinine and blood urea nitrogen). Dogs with proteinuria but without azotemia or hypoalbuminemia were permitted, as this is a suspected consequence of HTG in the breed [58]. Information on the primary diet(s) fed was collected and used to calculate dietary fat on a g/100 kcal basis. Dogs were all examined by one of the authors (E.F.) prior to having knowledge of HTG status and were given a body condition score on an ordinal scale of 1–9 (1 = emaciated, 9 = obese). Sex (male versus female) proportions were compared between case and control groups using a Fisher’s exact test. Age and dietary fat were compared between case and control groups with Student’s t-tests. Body condition score was compared between groups with a Wilcoxon rank-sum test. Statistical tests were run in R software (R version 4.2.2., www.r-project.org) [59].

Peripheral blood RNA was extracted using the Tempus Spin RNA Isolation Reagent Kit (ThermoFisher Scientific, Wilmington, DE). Quantity and quality of RNA was determined using the Nanodrop 8000 (ThermoFisher Scientific, Wilmington, DE), RiboGreen (Life Technologies, Carlsbad, CA), and the 2100 BioAnalyzer (Agilent, Santa Clara, CA). After quality control, samples underwent library preparation using the SMARTer Stranded Total RNA-Seq Kit v2, Pico Input Mammalian (Takar Bio, Mountain View, CA). This kit removes ribosomal RNA. RNA sequencing was performed using the NovaSeq 6000 S4 flow cell which produced 2 x 150bp paired end reads (Illumina, San Diego, Ca) at a targeted depth of 50 million reads per sample. RNA-Seq data was processed using PURR, a pipeline housed within the Collection of Hierarchical UMII-RIS Pipelines (CHURP) at the University of Minnesota [60]. CHURP analysis is provided as part of the RNA-Seq package run through the University of Minnesota Genomics Center. Raw reads were summarized using FastQC [61] and trimmed to remove low quality sequences using Trimmomatic [62].

Reads were aligned to the Dog10K_Boxer_Tasha/canFam6 canine reference genome assembly using HISAT2 [63]. Alignments were cleaned and name-sorted by feature. Features were only counted if both mates of a read pair mapped. Ten samples (5 cases and 5 controls) had previously undergone extraction and library preparation for a pilot analysis. Though these libraries were re-sequenced with the remaining new samples to avoid batch effects, a batch effect was still identifiable on a PCA plot. To correct for this, batch was included as a covariate in subsequent analyses. Raw read counts were used to perform differential expression analysis using the DESeq2 software package implemented in R [64]. Raw p-values were corrected for multiple testing using the Benjamini-Hochberg procedure, the default correction used by DESeq2 software, and statistical significance was defined as P_adj_ ≤ 0.05. Data visualization was performed using the plotPCA() function in DESeq2 and ComplexHeatmap package version 2.4.3 implemented in R [65] with hierarchical clustering turned on. Prior to final data visualization, batch effect was removed from the data using the removeBatchEffect() function implemented in ‘limma’ R package version 3.19 [66].

Because our conclusions are based on a handful of differentially expressed genes representing pro- and anti-thrombotic processes, we chose to perform RT-qPCR to confirm that the expression differences observed among the top two DEGs were reproducible. iScript Reverse Transcription Supermix was used for cDNA synthesis from 500 ng of total RNA following packages instructions (Bio-Rad Laboratories, Hercules, CA). Previously RT-qPCR validated genes (*GUSB* and *HNRNPH1* [67]), and corresponding published primers were selected as housekeeping genes. Exon spanning primers for *SERPINE1* and *AGHGAP29* were designed in Primer3Plus [68] (S5 Table in S1 File). Primers were optimized for Tm, and all reactions for a given sample were run in triplicate utilizing iTaq Universal SYBR Green Supermix according to package instructions (Bio-Rad Laboratories, Hercules, CA) with an annealing temperature of 61C on the Bio-Rad CFX96 Touch Real-Time PCR Detection System. Amplification efficiency from standard curves was between 106.2–129.8% as calculated by CFX Maestro. The 2^-(ΔΔCq) method was used to calculate relative fold expression between cases and controls. Significance of observed expression differences was determined using a Welch 2-sample T-test of ΔCq values performed in R.

To identify biological pathways that may be altered in the HTG disease state, pathway analysis of differentially expressed genes was performed using Enrichr [69–71]. Reported results are from the Reactome [72] and BioPlanet [73] databases. Reactome was chosen because it is a curated and peer-reviewed pathway database that is actively maintained, with the most recent update in 2022. BioPlanet is curated and maintained by scientists at the NIH National Center for Advancing Translational Sciences. It includes manually curated pathways and incorporates data from multiple pathway resources, including but not limited to Reactome, KEGG, BioCarta, and NCI-Nature; its most recent update was in 2019. Both databases were used to provide a comprehensive assessment of pathways that may be represented among differentially expressed genes. Due to the large number of pathways that were identified, we imposed a P_adj_<0.01 cutoff for statistical significance.

## Supporting information

S1 File (DOCX)
