# Supplementary material for: Whole blood gene expression analysis of spontaneous hypertriglyceridemia in dogs suggests an underlying pro-thrombotic process
Source: PLoS One. 2024 Nov 12;19(11):e0313343. doi: 10.1371/journal.pone.0313343 (PMC11556679; doi:10.1371/journal.pone.0313343)
Supplement: S1 File — (DOCX) [file pone.0313343.s001.docx]

| **HTG_ID** | **Trimmed Reads** | **2xReads** | **Assigned** | **Unassigned (Ambiguity)** | **Total Mapped Reads** | **Primary%** | **Secondary%** |
| --- | --- | --- | --- | --- | --- | --- | --- |
| Case_01 | 66423995 | 132847990 | 32,444,717 | 83,166 | 47,062,714 | 0.69 | 0.002 |
| Case_02 | 57840336 | 115680672 | 26,151,505 | 70,634 | 43,399,783 | 0.60 | 0.002 |
| Case_03 | 59812888 | 119625776 | 27,946,274 | 63,281 | 47,657,119 | 0.59 | 0.001 |
| Case_04 | 67938968 | 135877936 | 28,578,753 | 90,967 | 54,204,952 | 0.53 | 0.002 |
| Case_05 | 67129125 | 134258250 | 27,976,646 | 113,367 | 52,808,703 | 0.53 | 0.002 |
| Case_06 | 53637599 | 107275198 | 23,842,026 | 90,584 | 39,777,066 | 0.60 | 0.002 |
| Case_07 | 58754773 | 117509546 | 25,193,543 | 102,746 | 42,726,990 | 0.59 | 0.002 |
| Case_08 | 64297820 | 128595640 | 27,939,144 | 84,952 | 46,371,387 | 0.60 | 0.002 |
| Case_09 | 52719160 | 105438320 | 23,095,099 | 83,509 | 39,233,093 | 0.59 | 0.002 |
| Case_10 | 65950185 | 131900370 | 32,185,581 | 90,391 | 53,457,804 | 0.60 | 0.002 |
| Case_11 | 52240219 | 104480438 | 23,422,544 | 59,519 | 43,478,828 | 0.54 | 0.001 |
| Case_12 | 94070029 | 188140058 | 41,799,066 | 127,430 | 76,735,983 | 0.54 | 0.002 |
| Case_13 | 56383668 | 112767336 | 24,334,597 | 83,084 | 43,924,418 | 0.55 | 0.002 |
| Control_01 | 49094687 | 98189374 | 21,785,523 | 52,932 | 38,949,969 | 0.56 | 0.001 |
| Control_02 | 55039988 | 110079976 | 23,036,928 | 82,228 | 42,611,262 | 0.54 | 0.002 |
| Control_03 | 52251068 | 104502136 | 24,536,864 | 97,792 | 33,912,450 | 0.72 | 0.003 |
| Control_04 | 105395044 | 210790088 | 44,516,660 | 122,136 | 89,578,156 | 0.50 | 0.001 |
| Control_05 | 62072040 | 124144080 | 27,380,361 | 68,576 | 50,788,088 | 0.54 | 0.001 |
| Control_06 | 103870010 | 207740020 | 46,536,103 | 175,717 | 86,209,804 | 0.54 | 0.002 |
| Control_07 | 57863273 | 115726546 | 23,495,920 | 55,869 | 48,196,637 | 0.49 | 0.001 |
| Control_08 | 52336813 | 104673626 | 20,737,614 | 50,817 | 43,048,566 | 0.48 | 0.001 |
| Control_09 | 55365716 | 110731432 | 24,921,876 | 63,519 | 47,610,427 | 0.52 | 0.001 |
| Control_10 | 64493673 | 128987346 | 26,673,450 | 75,976 | 52,975,105 | 0.50 | 0.001 |
| Control_11 | 85632823 | 171265646 | 36,286,592 | 103,532 | 71,571,498 | 0.51 | 0.001 |
| Control_12 | 53841269 | 107682538 | 25,414,733 | 59,884 | 42,565,447 | 0.60 | 0.001 |
| Control_13 | 58970880 | 117941760 | 25,214,105 | 75,460 | 45,965,813 | 0.55 | 0.002 |
| Control_14 | 51668574 | 103337148 | 21,543,591 | 72,021 | 39,142,286 | 0.55 | 0.002 |
| Control_15 | 53865665 | 107731330 | 22,330,148 | 70,078 | 42,927,847 | 0.52 | 0.002 |
| Control_16 | 59641508 | 119283016 | 25,330,473 | 81,403 | 46,952,733 | 0.54 | 0.002 |
| Control_17 | 56953137 | 113906274 | 23,823,069 | 60,353 | 46,124,133 | 0.52 | 0.001 |
| Control_18 | 54189210 | 108378420 | 24,019,778 | 55,168 | 44,334,879 | 0.54 | 0.001 |
| **Averages** | **62,894,972.35** | **125789945** | **27,499,783** | **82,809** | **49,816,256.13** | **0.56** | **0.002** |
|  |  |  |  |  | **%** | **55.71** | **0.168** |

**S1 Table. Individual alignment statistics from RNA sequencing**

Trimmed Reads = R1 and R2 readcount after trimming to remove low-quality sequences; 2xReads = Trimmed reads multiplied by 2; Assigned = reads assigned to a feature; Unassigned (Ambiguity) = multiply mapped reads; Total mapped reads = all reads mapped to a feature; Primary% = Assigned/Total reads mapped; Secondary%=Unassigned/Total reads mapped.

**S2 Table. Genes that are differentially expressed between Miniature Schnauzer dogs with and without hypertriglyceridemia**

| **Gene** | **baseMean** | **log2FoldChange** | **lfcSE** | **stat** | **pvalue** | **padj** |
| --- | --- | --- | --- | --- | --- | --- |
| *ARHGAP29* | 73.3 | 2.02 | 0.34 | 5.90 | 3.69E-09 | 5.20E-05 |
| *SERPINE1* | 31.9 | 2.43 | 0.45 | 5.34 | 9.19E-08 | 4.40E-04 |
| *ARHGAP21* | 569.9 | 0.51 | 0.10 | 5.29 | 1.24E-07 | 4.40E-04 |
| *SHANK3* | 126.3 | 1.60 | 0.30 | 5.29 | 1.25E-07 | 4.40E-04 |
| *MMRN1* | 606.5 | 1.14 | 0.22 | 5.14 | 2.75E-07 | 7.77E-04 |
| *ST6GALNAC4* | 1524.7 | 0.65 | 0.13 | 5.06 | 4.13E-07 | 9.71E-04 |
| *FZD7* | 40.5 | 1.21 | 0.24 | 4.99 | 5.94E-07 | 1.20E-03 |
| *STON2* | 3905.2 | 0.66 | 0.14 | 4.78 | 1.73E-06 | 2.75E-03 |
| *RTN4* | 1690.7 | 0.21 | 0.04 | 4.78 | 1.76E-06 | 2.75E-03 |
| *SLPI* | 17.5 | 1.91 | 0.41 | 4.64 | 3.46E-06 | 4.88E-03 |
| *PTGS1* | 1789.2 | 0.69 | 0.15 | 4.58 | 4.70E-06 | 6.03E-03 |
| *BANF1* | 603.4 | 0.41 | 0.09 | 4.55 | 5.42E-06 | 6.38E-03 |
| *CTSG* | 27.5 | 1.55 | 0.34 | 4.53 | 5.88E-06 | 6.39E-03 |
| *ALDH2* | 1142.3 | 0.58 | 0.13 | 4.50 | 6.66E-06 | 6.71E-03 |
| *DUSP26* | 2578.6 | 0.53 | 0.12 | 4.44 | 8.91E-06 | 7.82E-03 |
| *TUBB1* | 14813.1 | 0.68 | 0.15 | 4.43 | 9.37E-06 | 7.82E-03 |
| *PHF14* | 1094.1 | -0.18 | 0.04 | -4.43 | 9.42E-06 | 7.82E-03 |
| *ZMAT4* | 32.5 | 1.12 | 0.25 | 4.42 | 1.00E-05 | 7.84E-03 |
| *ANO6* | 4308.8 | 0.51 | 0.12 | 4.39 | 1.14E-05 | 8.48E-03 |
| *CISH* | 897.0 | 0.66 | 0.15 | 4.38 | 1.20E-05 | 8.49E-03 |
| *LOC611083* | 468.8 | 0.81 | 0.19 | 4.36 | 1.28E-05 | 8.62E-03 |
| *THBS1* | 29020.5 | 0.75 | 0.17 | 4.35 | 1.35E-05 | 8.62E-03 |
| *C1QTNF6* | 43.8 | 1.37 | 0.32 | 4.32 | 1.57E-05 | 8.62E-03 |
| *MGAT4B* | 2055.3 | 0.38 | 0.09 | 4.31 | 1.61E-05 | 8.62E-03 |
| *CD34* | 24.9 | 1.09 | 0.25 | 4.31 | 1.65E-05 | 8.62E-03 |
| *EGF* | 2261.3 | 0.64 | 0.15 | 4.31 | 1.66E-05 | 8.62E-03 |
| *SEPTIN5* | 574.4 | 0.73 | 0.17 | 4.31 | 1.67E-05 | 8.62E-03 |
| *GCK* | 72.2 | 0.91 | 0.21 | 4.30 | 1.71E-05 | 8.62E-03 |
| *ENDOD1* | 2534.3 | 0.56 | 0.13 | 4.26 | 2.05E-05 | 9.84E-03 |
| *CHIC2* | 1193.1 | -0.33 | 0.08 | -4.25 | 2.13E-05 | 9.84E-03 |
| *GP1BB* | 1261.8 | 0.93 | 0.22 | 4.25 | 2.16E-05 | 9.84E-03 |
| *TRNT1* | 1055.5 | -0.43 | 0.10 | -4.22 | 2.50E-05 | 1.08E-02 |
| *ANKRD33B* | 765.8 | 0.54 | 0.13 | 4.21 | 2.53E-05 | 1.08E-02 |
| *NLK* | 2497.8 | 0.51 | 0.12 | 4.14 | 3.46E-05 | 1.36E-02 |
| *LOC111096422* | 17.7 | 1.68 | 0.41 | 4.14 | 3.49E-05 | 1.36E-02 |
| *SCIN* | 17.1 | 1.73 | 0.42 | 4.14 | 3.51E-05 | 1.36E-02 |
| *MAPK6* | 1109.4 | 0.61 | 0.15 | 4.13 | 3.56E-05 | 1.36E-02 |
| *SAMD12* | 80.4 | 0.76 | 0.18 | 4.13 | 3.67E-05 | 1.36E-02 |
| *ITGB3* | 10306.3 | 0.70 | 0.17 | 4.12 | 3.86E-05 | 1.39E-02 |
| *MPO* | 60.1 | 2.42 | 0.59 | 4.10 | 4.06E-05 | 1.39E-02 |
| *PADI3* | 211.5 | 0.72 | 0.18 | 4.10 | 4.09E-05 | 1.39E-02 |
| *LOC119866377* | 4586.5 | 0.56 | 0.14 | 4.10 | 4.14E-05 | 1.39E-02 |
| *LOC102153462* | 60.7 | 0.53 | 0.13 | 4.09 | 4.33E-05 | 1.41E-02 |
| *SIAE* | 195.6 | 0.52 | 0.13 | 4.08 | 4.54E-05 | 1.41E-02 |
| *ZCCHC3* | 511.1 | -0.31 | 0.08 | -4.08 | 4.56E-05 | 1.41E-02 |
| *GNAZ* | 1118.9 | 0.77 | 0.19 | 4.08 | 4.58E-05 | 1.41E-02 |
| *ALAD* | 347.9 | 0.54 | 0.13 | 4.07 | 4.75E-05 | 1.42E-02 |
| *CHST9* | 27.9 | 1.18 | 0.30 | 3.97 | 7.14E-05 | 2.10E-02 |
| *AGL* | 836.5 | 0.39 | 0.10 | 3.94 | 8.24E-05 | 2.37E-02 |
| *PINK1* | 851.8 | 0.42 | 0.11 | 3.93 | 8.59E-05 | 2.42E-02 |
| *PELO* | 193.0 | -0.48 | 0.12 | -3.92 | 8.88E-05 | 2.45E-02 |
| *TUBE1* | 121.9 | -0.49 | 0.13 | -3.92 | 9.03E-05 | 2.45E-02 |
| *ITGA2B* | 14000.1 | 0.72 | 0.18 | 3.91 | 9.29E-05 | 2.45E-02 |
| *LOC106559158* | 82.8 | -0.51 | 0.13 | -3.91 | 9.39E-05 | 2.45E-02 |
| *EPOR* | 263.3 | 0.74 | 0.19 | 3.89 | 9.90E-05 | 2.50E-02 |
| *TMEM117* | 446.3 | 0.62 | 0.16 | 3.89 | 9.99E-05 | 2.50E-02 |
| *LOC102156563* | 20.8 | -0.86 | 0.22 | -3.89 | 0.00010083 | 2.50E-02 |
| *CAPNS1* | 4862.1 | 0.33 | 0.08 | 3.88 | 0.00010634 | 2.59E-02 |
| *TSPAN9* | 361.8 | 0.70 | 0.18 | 3.87 | 0.0001103 | 2.64E-02 |
| *BCR* | 1751.8 | 0.71 | 0.18 | 3.86 | 0.0001153 | 2.71E-02 |
| *AMOTL2* | 29.1 | 1.01 | 0.26 | 3.83 | 0.00012877 | 2.92E-02 |
| *TXLNA* | 1496.0 | -0.22 | 0.06 | -3.83 | 0.00013012 | 2.92E-02 |
| *DGKH* | 2687.0 | 0.58 | 0.15 | 3.83 | 0.0001303 | 2.92E-02 |
| *CCDC57* | 919.6 | -0.34 | 0.09 | -3.80 | 0.00014218 | 3.13E-02 |
| *LOC111094232* | 36.1 | 0.69 | 0.18 | 3.79 | 0.00014855 | 3.23E-02 |
| *GSN* | 6015.0 | 0.46 | 0.12 | 3.79 | 0.0001526 | 3.26E-02 |
| *HCAR2* | 72.5 | 1.17 | 0.31 | 3.78 | 0.00015716 | 3.30E-02 |
| *TBC1D7* | 399.1 | 0.41 | 0.11 | 3.77 | 0.00016106 | 3.30E-02 |
| *PRKG1* | 198.1 | 0.97 | 0.26 | 3.77 | 0.00016338 | 3.30E-02 |
| *VPS41* | 1327.6 | 0.19 | 0.05 | 3.77 | 0.00016374 | 3.30E-02 |
| *DCSTAMP* | 1599.0 | 0.51 | 0.14 | 3.76 | 0.00016745 | 3.31E-02 |
| *RBPMS2* | 1843.2 | 0.56 | 0.15 | 3.75 | 0.00017513 | 3.31E-02 |
| *LOC111091261* | 575.0 | -0.50 | 0.13 | -3.75 | 0.00017704 | 3.31E-02 |
| *MERTK* | 18.9 | 1.47 | 0.39 | 3.75 | 0.00017741 | 3.31E-02 |
| *SRSF10* | 1915.7 | -0.22 | 0.06 | -3.75 | 0.00017831 | 3.31E-02 |
| *TMEM156* | 411.0 | -0.46 | 0.12 | -3.74 | 0.00018423 | 3.31E-02 |
| *TMEFF2* | 316.5 | 0.80 | 0.21 | 3.74 | 0.00018491 | 3.31E-02 |
| *LOC106558720* | 396.0 | -1.48 | 0.40 | -3.74 | 0.00018521 | 3.31E-02 |
| *NCKAP1* | 927.4 | 0.52 | 0.14 | 3.74 | 0.00018699 | 3.31E-02 |
| *METTL7A* | 3346.3 | 0.56 | 0.15 | 3.74 | 0.00018771 | 3.31E-02 |
| *UBE2D2* | 740.4 | -0.18 | 0.05 | -3.70 | 0.00021399 | 3.69E-02 |
| *CREB3L1* | 117.5 | 0.86 | 0.23 | 3.70 | 0.00021547 | 3.69E-02 |
| *TTYH3* | 3465.2 | 0.41 | 0.11 | 3.70 | 0.00021681 | 3.69E-02 |
| *VCL* | 7216.2 | 0.50 | 0.14 | 3.69 | 0.00022258 | 3.74E-02 |
| *TRA2A* | 1397.0 | -0.23 | 0.06 | -3.68 | 0.00022961 | 3.80E-02 |
| *LOC119871221* | 60.4 | -1.63 | 0.44 | -3.68 | 0.00023177 | 3.80E-02 |
| *FBXO38* | 1068.8 | -0.17 | 0.05 | -3.66 | 0.00024929 | 4.01E-02 |
| *LOC119870078* | 101.6 | -0.51 | 0.14 | -3.66 | 0.00024993 | 4.01E-02 |
| *KDR* | 425.8 | 0.48 | 0.13 | 3.65 | 0.00026674 | 4.23E-02 |
| *LOC106558650* | 599.9 | -0.39 | 0.11 | -3.64 | 0.00027058 | 4.24E-02 |
| *ZSCAN20* | 382.2 | -0.22 | 0.06 | -3.63 | 0.00027911 | 4.33E-02 |
| *VSIG10* | 171.2 | 0.51 | 0.14 | 3.63 | 0.0002835 | 4.35E-02 |
| *LTBP1* | 1116.7 | 0.56 | 0.16 | 3.62 | 0.00029082 | 4.41E-02 |
| *SDF4* | 2297.8 | 0.34 | 0.09 | 3.62 | 0.00029479 | 4.41E-02 |
| *LOC102151716* | 60.3 | 0.95 | 0.26 | 3.62 | 0.00029761 | 4.41E-02 |
| *ATP7B* | 42.3 | 0.67 | 0.19 | 3.61 | 0.00030056 | 4.41E-02 |
| *NCK2* | 4743.7 | 0.39 | 0.11 | 3.61 | 0.0003055 | 4.41E-02 |
| *PLS1* | 46.9 | 1.11 | 0.31 | 3.61 | 0.00030902 | 4.41E-02 |
| *TXLNB* | 1082.1 | 0.47 | 0.13 | 3.61 | 0.00030931 | 4.41E-02 |
| *ITGB1* | 8735.6 | 0.34 | 0.09 | 3.60 | 0.00032418 | 4.57E-02 |
| *CLCN3* | 611.6 | 0.35 | 0.10 | 3.58 | 0.00034183 | 4.75E-02 |
| *ANK2* | 136.5 | 0.78 | 0.22 | 3.58 | 0.00034509 | 4.75E-02 |
| *SLC24A3* | 141.9 | 0.67 | 0.19 | 3.58 | 0.00034766 | 4.75E-02 |
| *LOC102156518* | 271.5 | 0.66 | 0.18 | 3.57 | 0.00035319 | 4.75E-02 |
| *LOC119873892* | 55.4 | -0.63 | 0.18 | -3.57 | 0.00035336 | 4.75E-02 |
| *NSG1* | 149.5 | 0.95 | 0.27 | 3.57 | 0.00036017 | 4.80E-02 |
| *GATA2* | 76.2 | 1.08 | 0.30 | 3.56 | 0.00037198 | 4.91E-02 |
| *F13A1* | 6623.8 | 0.59 | 0.17 | 3.55 | 0.00038081 | 4.91E-02 |
| *CCAR1* | 3456.3 | -0.14 | 0.04 | -3.55 | 0.0003816 | 4.91E-02 |
| *HECA* | 5478.4 | -0.17 | 0.05 | -3.55 | 0.00038273 | 4.91E-02 |

BaseMean= mean of normalized counts for all samples. lfcSE= standard error of log 2 fold change. Stat= Wald test statistic, Pvalue=Wald test P-value; padj= Benjamini-Hochberg adjusted p-values.

**S3 Table. Reactome Pathways identified through EnrichR analysis**

| **Term** | **Overlap** | **P-value** | **Adjusted P-value** | **Odds Ratio** | **Combined Score** | **Genes** |
| --- | --- | --- | --- | --- | --- | --- |
| Platelet Degranulation R-HSA-114608 | 9/125 | 2.97E-08 | 7.40E-06 | 15.19 | 263.27 | MMRN1;EGF;ITGB3;SERPINE1;ITGA2B;  ENDOD1;F13A1;THBS1;VCL |
| Response To Elevated Platelet Cytosolic Ca2+ R-HSA-76005 | 9/130 | 4.18E-08 | 7.40E-06 | 14.56 | 247.35 | MMRN1;EGF;ITGB3;SERPINE1;ITGA2B;  ENDOD1;F13A1;THBS1;VCL |
| Platelet Activation, Signaling And Aggregation R-HSA-76002 | 10/254 | 1.41E-06 | 1.66E-04 | 8.05 | 108.48 | MMRN1;EGF;ITGB3;SERPINE1;ITGA2B;  ENDOD1;F13A1;THBS1;VCL;DGKH |
| Hemostasis R-HSA-109582 | 14/576 | 3.34E-06 | 2.96E-04 | 5.02 | 63.24 | ITGB1;EGF;ITGB3;SERPINE1;ITGA2B;  ENDOD1;F13A1;GATA2;MERTK;THBS1;  MMRN1;VCL;PRKG1;DGKH |
| Integrin Cell Surface Interactions R-HSA-216083 | 5/66 | 3.14E-05 | 1.96E-03 | 15.48 | 160.49 | ITGB1;ITGB3;ITGA2B;KDR;THBS1 |
| Extracellular Matrix Organization R-HSA-1474244 | 9/291 | 3.32E-05 | 1.96E-03 | 6.20 | 63.90 | ITGB1;CAPNS1;ITGB3;SERPINE1;  ITGA2B;KDR;CTSG;LTBP1;THBS1 |
| Syndecan Interactions R-HSA-3000170 | 3/20 | 1.72E-04 | 7.63E-03 | 32.78 | 284.03 | ITGB1;ITGB3;THBS1 |
| Signal Transduction By L1 R-HSA-445144 | 3/20 | 1.72E-04 | 7.63E-03 | 32.78 | 284.03 | ITGB1;ITGB3;ITGA2B |
| ECM Proteoglycans R-HSA-3000178 | 4/55 | 2.38E-04 | 9.36E-03 | 14.68 | 122.47 | ITGB1;ITGB3;ITGA2B;SERPINE1 |

**S4 Table. BioPlanet pathways identified through EnrichR analysis**

| Term | Overlap | P-value | Adjusted P-value | Odds Ratio | Combined Score | Genes |
| --- | --- | --- | --- | --- | --- | --- |
| Response to elevated platelet cytosolic calcium | 8/83 | 1.81E-08 | 7.67E-06 | 20.72 | 369.38 | MMRN1;EGF;ITGB3;ITGA2B;SERPINE1;  F13A1;THBS1;VCL |
| Platelet activation, signaling and aggregation | 10/205 | 1.98E-07 | 4.06E-05 | 10.10 | 155.90 | MMRN1;GP1BB;EGF;ITGB3;SERPINE1;  ITGA2B;F13A1;THBS1;VCL;DGKH |
| Hemostasis pathway | 14/468 | 2.88E-07 | 4.06E-05 | 6.24 | 94.03 | ITGB1;GP1BB;EGF;ITGB3;SERPINE1;  ITGA2B;F13A1;GATA2;MERTK;THBS1;  MMRN1;VCL;PRKG1;DGKH |
| Actin cytoskeleton regulation | 9/226 | 4.42E-06 | 4.68E-04 | 8.08 | 99.59 | ITGB1;NCKAP1;SCIN;GSN;EGF;ITGB3;  ITGA2B;MAPK6;VCL |
| Focal adhesion | 9/233 | 5.67E-06 | 4.79E-04 | 7.82 | 94.51 | ITGB1;EGF;ITGB3;ITGA2B;KDR;MAPK6;  PELO;THBS1;VCL |
| Integrin-mediated cell adhesion | 6/100 | 1.89E-05 | 1.33E-03 | 12.15 | 132.14 | ITGB1;CAPNS1;ITGB3;ITGA2B;  MAPK6;VCL |
| Mu-calpain pathway | 3/15 | 7.02E-05 | 3.83E-03 | 46.44 | 444.20 | ITGB1;CAPNS1;ITGB3 |
| TGF-beta signaling pathway | 7/185 | 7.38E-05 | 3.83E-03 | 7.53 | 71.61 | ITGB1;EGF;ITGB3;SERPINE1;MAPK6;  LTBP1;THBS1 |
| Urokinase-type plasminogen activator (uPA) and uPAR-mediated signaling | 4/42 | 8.25E-05 | 3.83E-03 | 19.71 | 185.36 | ITGB1;ITGB3;SERPINE1;CTSG |
| Beta-3 integrin cell surface interactions | 4/43 | 9.06E-05 | 3.83E-03 | 19.21 | 178.80 | ITGB3;ITGA2B;KDR;THBS1 |
| ECM-receptor interaction | 5/84 | 1.00E-04 | 3.86E-03 | 11.94 | 109.94 | ITGB1;GP1BB;ITGB3;ITGA2B;THBS1 |
| Hematopoietic cell lineage | 5/88 | 1.25E-04 | 4.41E-03 | 11.36 | 102.11 | GP1BB;ITGB3;ITGA2B;CD34;EPOR |
| S1P/S1P1 pathway | 3/21 | 2.00E-04 | 6.52E-03 | 30.95 | 263.59 | GNAZ;ITGB3;KDR |

**S1 Fig. Plotted normalized gene counts of top 10 differentially expressed genes between Miniature Schnauzer dogs with (cases) and without (controls) hypertriglyceridemia**

**
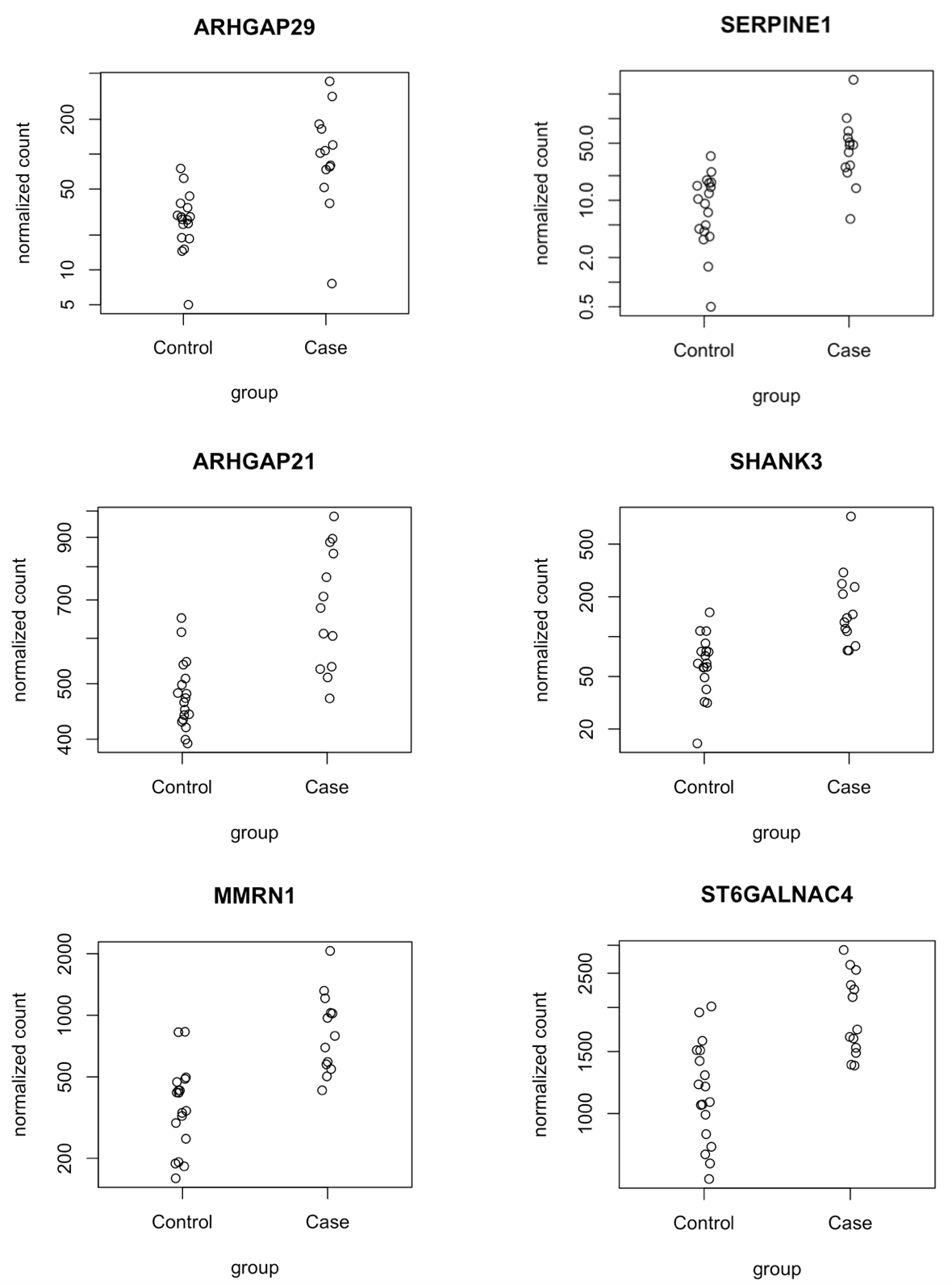
**

**
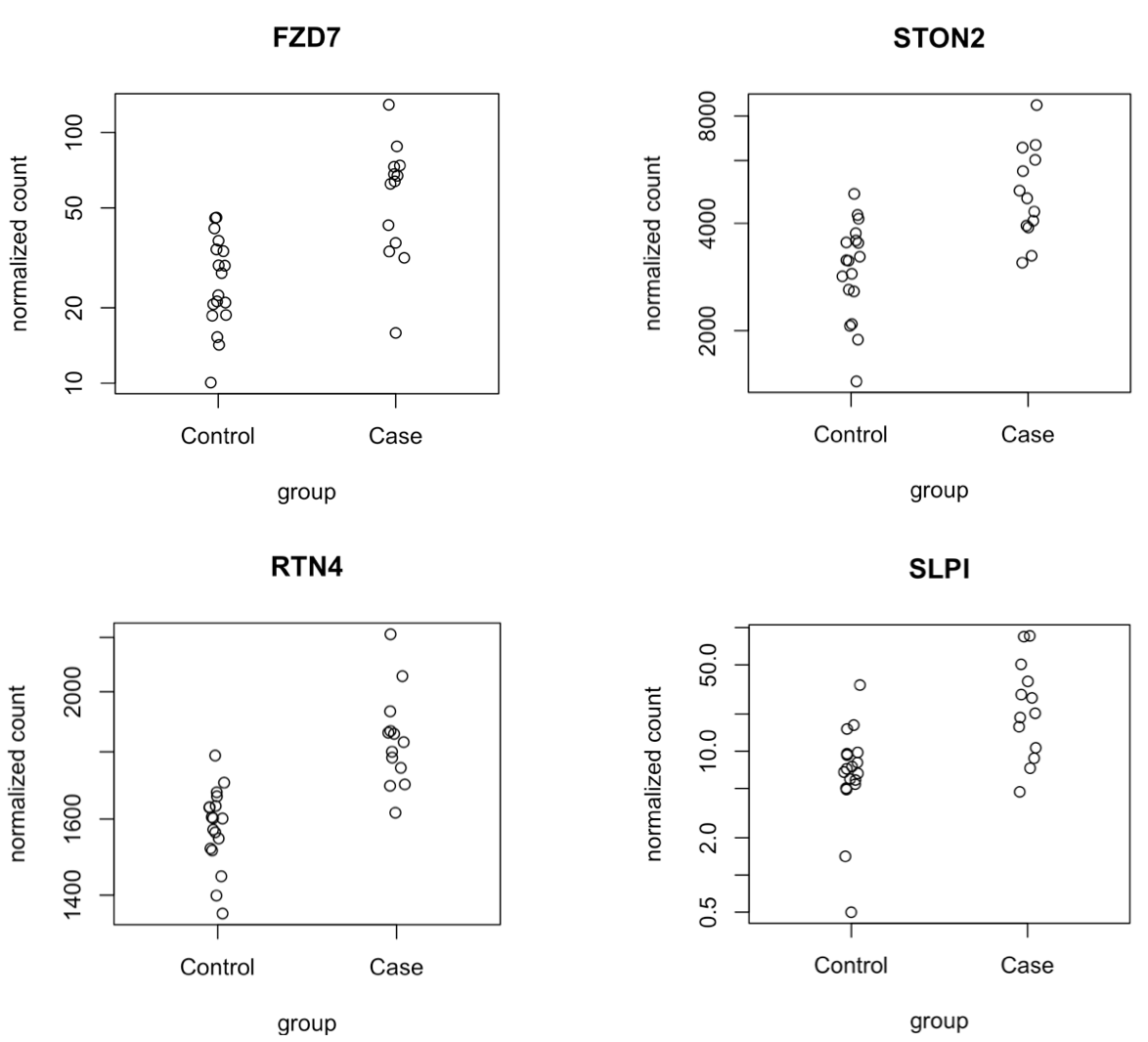
**

**S2 Fig. Plotted ΔCq from RT-qPCR of top differentially expressed genes *ARHGAP29* and *SERPINE1* in Miniature Schnauzer dogs with (cases) or without (controls) hypertriglyceridemia.**

**
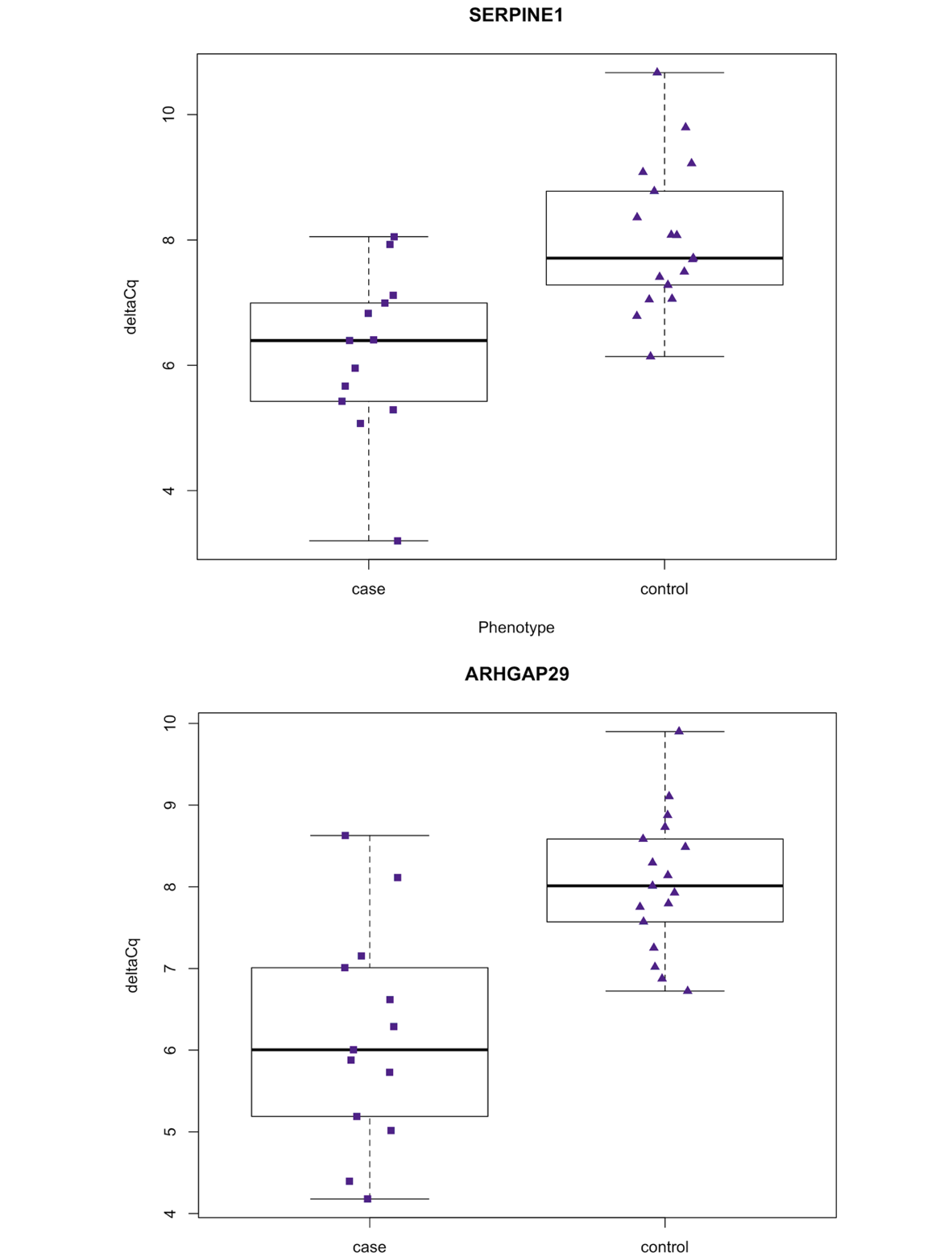
**

| **Target genes** |  |  |
| --- | --- | --- |
| SEPRINE1 cDNA F | CTGGTTCTGCCTAAGTTCTCC | 129 bp |
| SERPINE1 cDNA R | CAGCACCTCTTGATTTGAAAG |  |
|  |  |  |
| ARHGAP29 cDNA F | ATTTGAGGAATCGGAACGAA | 100 bp |
| ARHGAP29 cDNA R | TGATTCAGGTTCTTGGTCTGC |  |
|  |  |  |
|  |  |  |
| **Housekeeping genes** |  |  |
| GUSB cDNA F | AGACGCTTCCAA/GTACCCC | 103 bp |
| GUSB cDNA R | AGGTGTGGTGTAGAGGAGCAC |  |
|  |  |  |
| HNRNPH1 cDNA F | CTCACTATGATCCACCACG | 151 bp |
| HNRNPH1 cDNA R | TAGCCTCCATAACCTCCAC |  |

**S5 Table. Primers used in confirmatory RT-qPCR for top genes *SERPINE1* and *ARHGAP29***
